# Novel tacrine–benzofuran hybrids as potential multi-target drug candidates for the treatment of Alzheimer’s Disease

**DOI:** 10.1080/14756366.2019.1689237

**Published:** 2019-11-25

**Authors:** Gaia Fancellu, Karam Chand, Daniel Tomás, Elisabetta Orlandini, Luca Piemontese, Diana F. Silva, Sandra M. Cardoso, Sílvia Chaves, M. Amélia Santos

**Affiliations:** a Centro de Química Estrutural, Instituto Superior Técnico, Universidade de Lisboa, Lisboa, Portugal;; b Department of Earth Sciences, University of Pisa, Pisa, Italy;; c Dipartimento di Farmacia–Scienze del Farmaco, Università degli Studi di Bari “Aldo Moro”, Bari, Italy;; d CNC–Center for Neuroscience and Cell Biology, Universidade de Coimbra, Coimbra, Portugal;; e Institute of Molecular and Cell Biology, Faculty of Medicine, Universidade de Coimbra, Coimbra, Portugal

**Keywords:** Alzheimer’s disease, multi-target drugs, tacrine-benzofuran hybrids, AChE inhibitors, metal chelators

## Abstract

Pursuing the widespread interest on multi-target drugs to combat Alzheimer´s disease (AD), a new series of hybrids was designed and developed based on the repositioning of the well-known acetylcholinesterase (AChE) inhibitor, tacrine (TAC), by its coupling to benzofuran (BF) derivatives. The BF framework aims to endow the conjugate molecules with ability for inhibition of AChE (bimodal way) and of amyloid-beta peptide aggregation, besides providing metal (Fe, Cu) chelating ability and concomitant extra anti-oxidant activity, for the hybrids with hydroxyl substitution. The new TAC-BF conjugates showed very good activity for AChE inhibition (sub-micromolar range) and good capacity for the inhibition of self- and Cu-mediated Aβ aggregation, with dependence on the linker size and substituent groups of each main moiety. Neuroprotective effects were also found for the compounds through viability assays of neuroblastoma cells, after Aβ_1-42_ induced toxicity. Structure-activity relationship analysis provides insights on the best structural parameters, to take in consideration for future studies in view of potential applications in AD therapy.

## GRAPHICAL ABSTRACT

**Figure:**
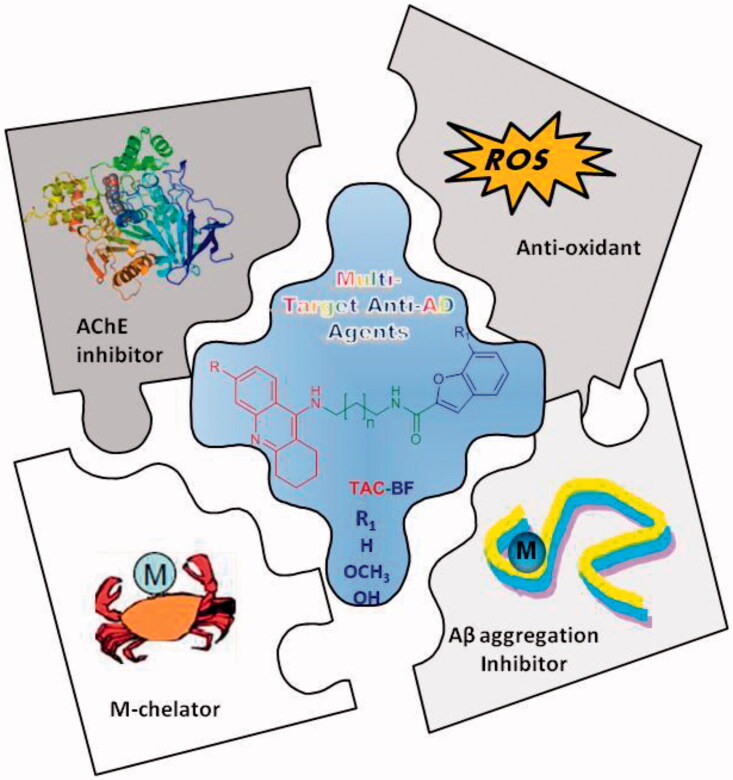

Design, synthesis and evaluation of new tacrine-benzofuran hybrids as multitargeting anti- Alzheimer’s disease agents; high AChE inhibition associated with other relevant properties; structure–activity relationship analysis

## 1. Introduction

Alzheimer’s Disease (AD) is one of the main causes of dementia among elderly people, whose prevalence by 2050 is expected to be 14 and 130 million people in Europe and worldwide, respectively^1^^,^^2^. It is characterised by progressive impairment of memory cognitive functions due to the degeneration of synapses and to the death of neurons, especially in hippocampus^3^. The main pathological hallmarks of AD are the presence of thick extracellular β-amyloid plaques (Aβ) and intra-neuronal neurofibrillary tangles (NFT)^4^, which compromise brain function due to the death of many neurons. Other relevant characteristics of the patient brains are the deficit of choline neurotransmitters, mostly acetylcholine due to its hydrolysis by acetylcholinesterase (AChE) in neuronal synapses, and also the metal dyshomeostasis associated with advanced oxidative processes. In spite of the huge amount of research aimed at understanding and to combat this pathology, there is no cure so far. Along the past two decades, several strategies have been followed bringing important contributions to AD drug design and therapy^5^. The inhibition of AChE appeared as the first approach and four AChE inhibitor drugs (tacrine, donepezil, galantamine and rivastigmine) were approved by the US FDA, although they can only lead to temporary symptom amelioration^6^. Despite enormous efforts in the recent past years from academia and pharmaceutical companies, several new molecules failed on clinical trials^7^.

Due to the multifactorial nature of this disease, the perspective of AD treatment with a single-target molecule has been recently changed with the emergence of multi-target anti-AD drug candidates, aimed at targeting the disease pathogenesis and get disease–modifying effects. This type of compounds can act on several disease targets, such as amyloid beta peptide (Aβ) aggregation, inhibition of AChE, modulation of metal dyshomeostasis and inhibition of MAO involved in the production of reactive oxygen species (ROS)^8–11^. Therefore, the world of hybrid molecules has been discovered and deepened, namely by repositioning and extra-functionalization of already approved drugs such as acetylcholinesterase inhibitors (AChEi). Tacrine (TAC) is the first single drug developed for AD treatment as AChE inhibitor. Although it was withdrawn from the market due to its hepatic toxicity at therapeutic doses, it has been, by far, the mostly used AChE inhibitory moiety in the development of multi-target anti-AD drugs^11–13^.

Continuing our recent efforts to discover novel multi-target directed ligands (MTDLs) for the treatment of AD, especially based on templates of already known AChEi drugs^14–16^, the current study is focused on a new set of tacrine-benzofuran (TAC-BF) hybrids, which have been developed and evaluated for their multiple properties as potential multi-target anti-AD agents. The naturally inspired benzofuran (BF) scaffold can be considered as a mimic of the indanone moiety of donepezil, and it has been recently applied in many natural-based compounds with important biological properties, even as anti-neurodegeneratives^15^^,^^17–19^. Particularly in this study, the new hybrids were designed to decide on the most adequate spacer length between both main molecular units, with aid from molecular docking simulations. Afterwards, the selected compounds were synthesised and tested for their physico-chemical properties, as anti-oxidant and metal chelation capacity, and biological properties (AChE inhibition, anti-Aβ aggregation); the effects of the compounds on cell viability and neuroprotection were also evaluated in neuroblastoma cells after Aβ_1-42_ induced toxicity.

## 2. Results and Discussion

### 2.1. Molecular design

**Figure 1.**
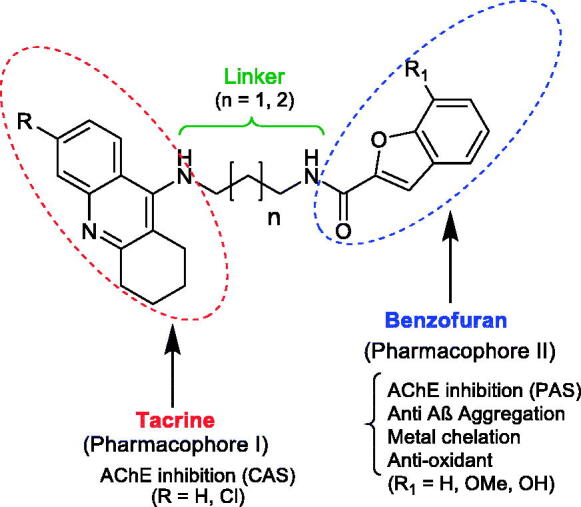
General structure of the tacrine-benzofuran (TAC-BF) hybrids under study.

The study of the new multifunctional hybrid compounds (Figure 1) involved a preliminary molecular design approach. Therefore, two main molecular moieties were selected, namely tacrine (TAC) and benzofuran (BF), to guarantee the hitting of at least two main pathophysiological targets of AD. TAC could assure the AChE inhibition; BF was selected to mimic the indanone role in donepezil (DNP), enabling a bimodal enzyme interaction for AChE, and also to inhibit the self-aggregation of Aβ peptide; the hydroxyl BF substituent (β-positioned to the oxygen atom of BF) can also endow the hybrids with metal (Fe, Cu) chelating capacity and concomitant extra anti-oxidant activity. Besides the selection of the main scaffolds and corresponding linkers, substituent groups were also introduced in each moiety, namely: at C6 of TAC, the chlorine atom, aimed at improving the AChE inhibitory capacity; at C7 of BF, the methoxyl and hydroxyl groups, with the goal of mimicking DNP indanone substituent groups as well as to provide metal chelation capacity, respectively. To achieve the bimodal interaction of the hybrids with AChE, through the binding to both the catalytic anionic site (CAS) and the peripheral anionic site (PAS), the size of the linker chain, connecting both main moieties, is of expected relevance. Based on our previous experience about TAC hybrids with dual-binding site^16^, alkyl linkers were selected with 3–4 methylene carbons (*n* = 1, 2), meaning a total spacer length of 5–6 atoms. Therefore, to get some insight on the effects of these structural variations on the binding pattern and binding affinity between the ligand and the receptor, molecular docking simulation appears as an important tool.

The model structure for the active site of AChE was obtained from the RCSB Protein Data Bank (PDB, entry 1ODC)^20^, particularly from the X-Ray structure of *Torpedo Californica* AChE (*Tc*AChE) complexed with the inhibitor (*N*-4′-quinolyl-*N′*-9″-(1″,2″,3″,4″-tetrahydroacridinyl)-1,8-diaminooctane), hereinafter named as the original ligand, which has structural similarities with our hybrids. Notwithstanding some recognised differences between the respective inhibitor-enzyme complexes, reported for human acetylcholine esterase, hAChE, and also of the electric ray (Torpedo californica) homologue, TcAChE, both enzymes are fairly conservative in terms of the main aminoacid residues that coat the active site gorge^21^, and so the herein described modelling study was performed with *Tc*AChE.

**Figure 2.**
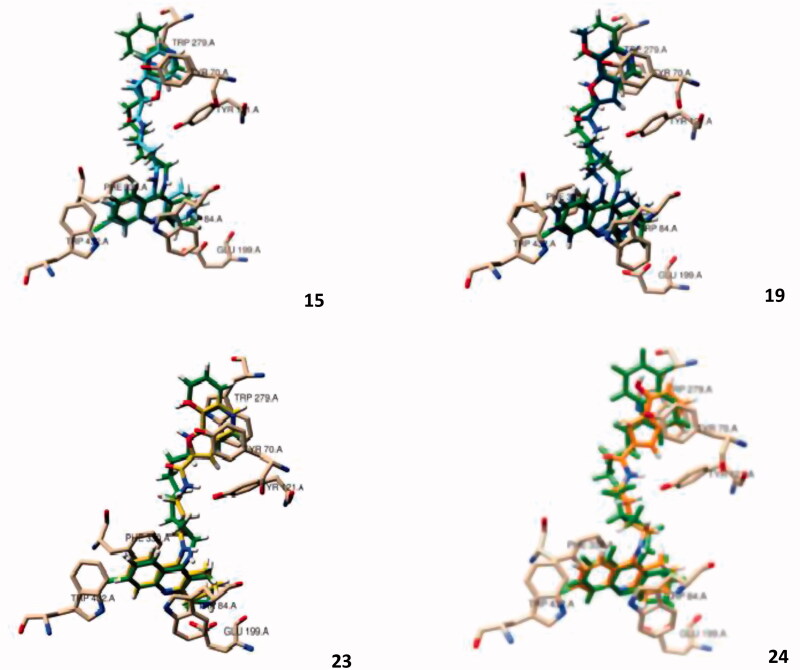
Molecular modelling of four representative compounds inside the binding site of AChE, superimposed with the original ligand (*N*-4′-quinolyl-*N*′-9″-(1″,2″,3″,4″-tetrahydroacridinyl)-1,8-diaminooctane), green Coloured. These compounds have the same TAC substituent (R = Cl), but different BF substituent (R_1_) and spacer length: **15** (turquoise Coloured; *n* = 1; R_1_ = H), **19** (dark blue Coloured; *n* = 1; R_1_ = -OCH_3_), **23** (yellow Coloured; *n* = 1; R_1_= -OH), **24** (orange Coloured; *n* = 2; R_1_ = H).

Analysis of the outcomes of docking results (Figure 2) shows a perfect insertion of the TAC moiety into the CAS, lined with the residues Trp84, Ph330, Glu199, with a good superimposition of the TAC of the hybrids and that of the original ligand. This is mainly due to their ability to establish π-π stacking binding interactions with the aromatic rings of Trp84 and Phe330 (see Figure 2), which can block the entrance of the substrate (ACh) and therefore avoid its hydrolysis by the catalytic triad (Ser200, His440, Glu327)^22^. On the other hand, the BF moiety, linked to TAC by the alkylamide spacers, seems also to be able to establish π-π interactions with PAS through Tyr70 and Trp279 residues. Both the alkyl-amidic chain linkers of the conjugates, with three or four methylene groups (*n* = 1, 2) in the alkyl chains, appear to be well accommodated along the hydrophobic channel, providing the adequate lengths for dual mode interaction inside the AChE gorge and also H-bond interactions between the amide-NH and the phenolic group of Tyr121, in accordance with previously reported results for other TAC hybrids^14^^,^^16^. Remarkably, the docking results show only small differences in the enzyme interaction with an apparent better interaction with shorter spacer (5 atoms in the tether chain; *n* = 1) than with the corresponding longer homologue (6 atoms in the tether chain; *n* = 2), (cf. **23** and **24**, Figure 2).

Regarding the effect of substituent groups, the chloro substitution at C6 of TAC is supposed to account for a better activity than the non-substituted analogues, due to the good fitting of the chlorine atom inside a hydrophobic pocket of *Tc*AChE formed by Trp432, Met436 and Ile439^23^, otherwise identically reported for the human AChE (*h*AChE)^24^, although, in Figure 2, only the first residue (Trp432) can be seen in a close distance from the chlorine atom. Regarding the BF moiety, the C7 substitution by methoxyl/hydroxyl groups aimed at enabling eventual new potential interactions into the PAS. Indeed, it is interesting to observe that the BF moiety is well accommodated into the PAS, where -OCH_3_/-OH seems to create H-bond interactions with the Tyr70, although the presence of the substituents may also cause a slight rearrangement of the whole structure inside this site, as compared with the non-substituted compounds. Overall, the molecular modelling studies gave some support to the predicted ability of the compounds for a bimodal interaction at CAS and PAS binding sites of AChE. Furthermore, BF is expected to bring other important contributions to the whole multifunctional activity of the studied hybrids (antioxidant activity, anti-Aβ aggregation and metal chelation), which will be object of study in the respective sections.

It should be mentioned that after the design and synthesis of the herein studied series of hybrids, a publication appeared also with TAC-BF hybrids^25^, although they have different linkers and do not include the same substituent groups, in particular the hydroxyl group of the benzofuran ring which is able to provide metal chelating capacity and additional anti-oxidant activity.

Comparison of the present docking simulations with those recently reported,^25^ for two other Tacrine-benzofuran hybrid analogues, indicates quite identical interaction within the CAS binding site, although interaction with PAS at the entrance of the gorge involves different aminoacid residues, attributable to the difference in the used crystal structure of the inhibitor-bound *Tc*AChE complexes and also to the much longer length of the linker for the two most closed analogues (from 3 to 6 carbon atom chain, in the present and the reported studies).

### 2.2. Chemistry

**Scheme 1.**
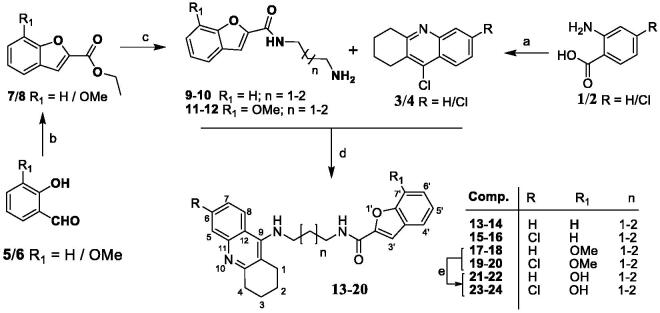
Synthesis of TAC-BF hybrids. Reagents and conditions: (a) cyclohexanone, POCl_3_, 180 °C, 3 h; (b) DMF, K_2_CO_3_ (1.2 eq), ethylbromoacetate (1.05 eq), 135-140 °C, 5–6 h; (c) diaminoalkane (3.0 eq), dry MeOH, overnight; (d) phenol, KI, 165–170 °C 35–60 min; (e) BCl_3_, TBAI, DCM, under N_2_ (−78 °C), 2 h.

The series of eight TAC-BF hybrids was prepared by following the synthetic strategy outlined in Scheme 1. It involved two independent sequential reaction steps leading to the preparation of the two main molecular moieties (TAC and BF), which were then coupled to each other. The TAC starting fragments, 9-chloro-1,2,3,4-tetrahydroacridines (**3/4**), were prepared from the coupling the commercially available anthranilic acid (**1)** or 2-amino-4-chlorobenzoic acid (**2)** with cyclohexanone in the presence of POCl_3_, as previously reported^26^. The synthesis of the BF derivatives involved a first condensation of the salicylaldehyde derivatives (**5/6)** (R_1_ = H/OMe) with ethylbromoacetate in dry DMF under reflux and basic conditions for substitution at the phenol group to obtain the intermediate esters, ethyl 2-phenoxyacetates (**5′/6′)**, which were subsequently cyclized by further heating at high temperature conditions to afford the corresponding intermediate BF-esters **(7/8).** Reaction of the diaminoalcane spacers with the benzofuran-2-ethylcarboxylates in methanol gave the corresponding *N*-(aminoalkyl)benzofuran-2-carboxamides (**9–12**). These aminoalkyl-benzofuran derivatives were finally attached at the 9-position of the TAC derivatives (**3**/**4**), by their reaction in the presence of phenol and a catalytic amount of potassium iodide, affording the final compounds (**13–20**). The metoxy-containing compounds (R_1_ = -OCH_3_, **18–20**) were further demethylated to obtain the corresponding derivatives with R_1_ = -OH (**21–24**). This reaction involved a standard acidic hydrolysis under very mild conditions, namely with a mixture of the Lewis acid boron trichloride (BCl_3_) and the catalyst tetra-*n*-butylammonium iodide (TBAI), in dry CH_2_Cl_2_, under N_2_ atmosphere and low temperature (−78 °C), following a protocol previously reported^27^^,^^28^.

### 2.3. Physico-chemical studies

#### 2.3.1. Radical scavenging activity

A selection of the newly synthesised hybrids was tested for their radical scavenging activity (anti-oxidant activity, EC_50_), based on their interaction with the 2,2-diphenyl-1-picrylhydrazyl (DPPH) free radical, as previously described^29^.

Analysis of the obtained results indicate for compound **20** a weak activity (EC_50_ >10^3^ µM), otherwise similar to that found for the reference compound, TAC. However, the introduction of the hydroxyl substituent in the BF moiety resulted in a considerable increase of the radical scavenging activity, though with still moderate EC_50_ values (434–480 µM, for **24** and **21**). Overall, the presence of the hydroxyl group increases the hybrid anti-oxidant activity, as previously anticipated, which can be attributed to their capacity for proton donation from the OH group.

#### 2.3.2. Metal complexation

It has been largely accepted that chelators interfere both in metal-induced Aβ aggregation/neurotoxicity and on metal homeostasis of AD brains. Among the hybrids with BF hydroxyl substituent, compound **21** was chosen as a representative model to analyse the concomitant chelating capacity towards the redox-active (Fe(III), Cu(II)) and Aβ-binding (Cu(II)) metal ions. Due to solubility reasons, these studies were performed in a mixed 25% (w/w) DMSO/water medium, In fact, DMSO was chosen because it is a widely used and well tolerated solvent in biological and cellular studies, the amounts of ligand (<7 µM) and DMSO (<1%) employed in culture media being quite low with concomitant no alterations observed in cells^30^.

**Figure 3.**
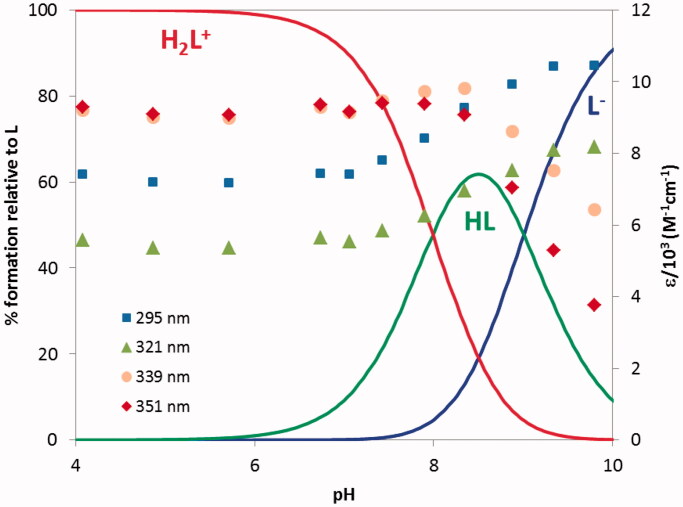
Species distribution curves for compound **21** along with molar extinction coefficients at the maximum absorption wavelengths (*C*_L_ = 4.0 × 1 0 ^−5 ^M).

The protonation constants of compound **21**, corresponding to the phenolic hydroxyl (log *K*_1_ = 9.01) and to the tacrine *N*-amine (log *K*_2_ = 7.99) groups, were determined by ultraviolet-visible (UV-vis) spectrophotometric titration (see Figure 3 and Table 1). Figure 3 shows that the absorvance maxima at 339 and 351 nm correspond to both species (H_2_L^+^ and HL), while those at 295 and 321 nm are attributed to the deprotonated (L^−^) form.

**Table 1.** Stepwise protonation constants for the ligand **21** and global formation constants^a^ for the corresponding Fe(III) and Cu(II)) complexes (*T* = 25.0 ± 0.1 °C, *I* = 0.1 M KCl, 25% w/w DMSO/water) as well as pM^b^ values.

Compound	M_m_H_h_L_l_	log *K*_i_	log $\beta_{{\text{Fe}}_{m}H_{h}L_{l}}$	log $\beta_{{\text{Cu}}_{m}H_{h}L_{l}}$
	(011)	9.01(1)		
	(021)	7.99(3)		
	(101)		–	10.87(6)
	(111)		22.90(6)	19.02(2)
	(102)		–	20.68(8)
	(112)		38.37(6)	–
	(103)		36.95(7)	–
	(1–11)		–	2.25(3)
	(1–22)		12.21(8)	**-**
	**pM**		**22.2**	**11.9**
	(011)	9.02(3)		
	(021)	8.18(5)		
	(031)	3.32(6)		
	(111)		**–**	17.27(5)
	(122)			34.38(6)
	(102)			20.14(8)
	**pM**			**11.1**
			

a $\beta_{M_{m}H_{h}L_{l}}$= [M_m_H_h_L_l_]/[M]^m^[H]^h^[L]^l^.

b pM = −log[M] at pH 7.4 (*C*_L_/*C*_M_ = 10, *C*_M_ = 1 0 ^−6 ^M).

c In 50/50 w/w DMSO/water^31^.

From analysis of Table 1 it is possible to conclude that the obtained log *K*_i_ values are in accordance with the values found, in 50% w/w DMSO/water medium, for the protonation of the phenolic oxygen (log *K*_1_ = 9.02 and the tacrine *N*-amine (log *K*_2_ = 8.18) of **TAC-BIM1**^31^.

The chelating ability of compound **21** towards the two metal ions under study (Fe(III) and Cu(II)) was evaluated on the basis of the global formation constants of the complexes, obtained by treatment of the UV-vis spectrophotometric data (see Table 1).

**Figure 4.**
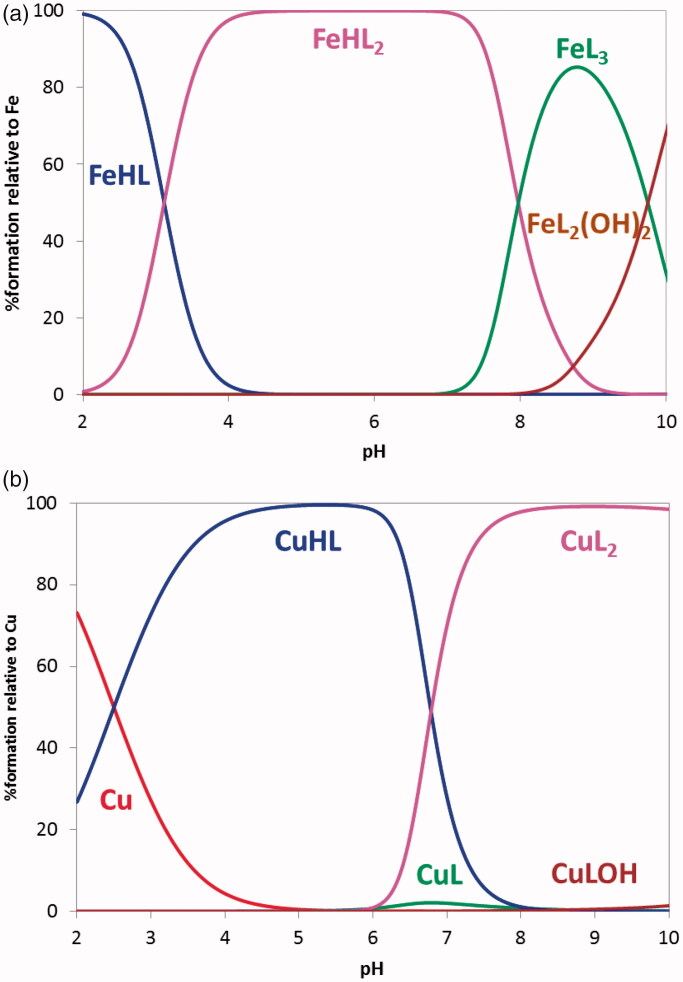
Species distribution curves for the systems (a) Fe(III)/**21** 1:3 and (b) Cu(II)/**21** 1:2 (*C*_L_ = 4.0 × 1 0 ^−5 ^M).

From Figure 4(a)) it is possible to observe that, under the working conditions, FeHL and FeHL_2_ are the predominant species up to pH ca 8 and above that pH FeL_3_ and FeL_2_(OH)_2_ are subsequently formed. Compound **21** is a strong chelator for iron (pFe = 22.2 at pH 7.4, *C*_L_/*C*_M_ = 10, *C*_M_ = 10^−6 ^M), that can be attributed to the *hard* donor atom (*O*,*O*) chelation core of the BF moiety and the formation of stable five-membered metal chelate rings.

Concerning copper chelation, Figure 4(b)) presents CuHL as the dominant species below pH 6.7 while above it, CuL_2_ is the predominant species. Compound **21** is a good copper chelator (pCu = 11.9), similarly to already found for the hydroxyphenyl-benzimidazole (BIM) derivatives (pCu = 10.7–11.1)^31^.

As a conclusion, the hydroxyl-containing derivatives of these TAC-BF compounds showed to be effective chelators of Fe(III) and Cu(II), well-known redox-active metal ions associated to AD.

### 2.4. Biological activity

#### 2.4.1. AChE inhibition

**Figure 5.**
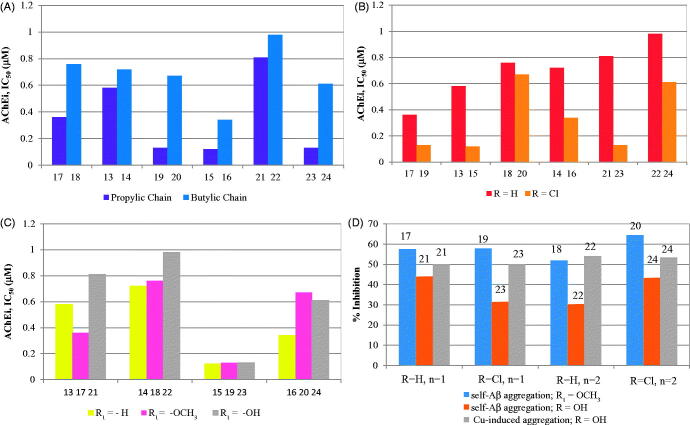
Graphical summary of the effect of different structural parameters on the inhibition of AChE (AChEi, IC_50_) and on the inhibition of Aβ aggregation (%): (A) size of alkylchain linker in AChEi: *n* = 1 (propylic chain, *n* = 2 (butylic chain); (B) substituents at C6 of TAC (R = H, Cl) in AChEi; (C) substituents at C7 of BF (R_1_ = H, -OCH_3_, -OH) in AChEi; (D) inhibition of Aβ aggregation (self- and Cu-induced).

Inhibition of *Tc*AChE by the new set of hybrids (**13–24**) was evaluated by using a previously described method^29^, based on an adaptation of the Ellman’s test^32^. The standard reference compound selected for these assays was tacrine (IC_50_ = 0.35 µM)^14^, which was also evaluated under our experimental conditions. The IC_50_ values obtained for AChE inhibition (AChEi) are depicted in Table 2. All the studied compounds showed good inhibitory activity, with IC_50_ values in submicromolar range, close or even inferior to that of the standard reference. A brief structure-activity relationship analysis was performed, to aid the comparison and understanding of the contribution of different structural variations in the inhibitory activity, namely the length of the spacer, as well as the presence of substituent groups in tacrine (H, Cl) and in benzofuran (-H, -OCH_3_, -OH), as illustrated on the graphics (A, B, C) of Figure 5.

**Table 2.** Summary of results for the biological assays of TAC-BF hybrids

	n	R	R_1_	AChE inhibition^a^ IC_50_ (µM)	Aβ aggregation inhibition^b,c^ (%)	
					Self-Aβ aggr	Cu-ind Aβ aggr
**13**	1	H	H	0.58	60.6	–
**14**	2	H	H	0.72	51.5	–
**15**	1	Cl	H	0.12	51.0	–
**16**	2	Cl	H	0.34	43.0	–
**17**	1	H	OCH_3_	0.36	57.5	–
**18**	2	H	OCH_3_	0.76	51.9	–
**19**	1	Cl	OCH_3_	0.13	57.8	–
**20**	2	Cl	OCH_3_	0.67	64.4	–
**21**	1	H	OH	0.81	44.0	50.1
**22**	2	H	OH	0.98	30.4	54.0
**23**	1	Cl	OH	0.13	31.5	52.5
**24**	2	Cl	OH	0.61	43.4	53.4
**TAC**	–	–	–	0.35	20	–

a The values are mean of five independent experiments ± SD; AChE from *Electric eel*.

b Inhibition of self-mediated Aβ_42_ aggregation (%) with or without copper (40 µM). The thioflavin-T fluorescence method was used, and the measurements were carried out in the presence of an inhibitor (80 µM).

c The values are the mean of two independent measurements in duplicate (SEM < 10%).

Graph **A** illustrates the effect of the linker length on the enzyme inhibitory capacity. Remarkably, the compounds with propylic chains evidenced higher AChE inhibitory activity than the butylic analogues, although the molecular modelling only indicated a subtle better accommodation of the shorter length inhibitors inside the enzyme active site. The compounds with longer linker may be forced to some structural distortions, namely in their interactions inside the lipophilic channel between CAS and PAS and even in the stacking interaction of BF with PAS. Interestingly, identical chain length effects were also found in other tacrine hybrids^16^. Graph **B** illustrates the effect of chlorine substitution in TAC, which led to improvement of the inhibitory activity of the hybrids, consistently with other results previously described^14^^,^^22^, although the major effect was shown for those with the propylic chain and OH substituent (cf. **21** and **23**). Graph **C** indicates that the BF substituents (-OCH_3_ or –OH) do not lead to activity improvement. Overall, the best AChEi activity was achieved for hybrids **15**, **19** and **23** with propylic chain linkers and, except **19,** as well as chloro substitution on TAC and no substitution on the BF moiety.

Although not measured under the framework of this paper, the inhibition of butylcholinesterase (BuChE) and the selectivity of both cholinesterases could be also of interest to be pursued in future evaluations.

#### 2.4.2. Inhibition of self- and Cu^2+^-induced Aβ_1-42_ aggregation

The Aβ amyloid plaques are one of the main hallmarks of AD and they result from over production and aggregation of β-amyloid peptide, which can accumulate outside the neurons, by self-mediated or Cu^2+^-induced process, interfering with the synaptic impairment, neurotransmission and memory^33^. Therefore, anti-AD drugs have been designed aimed to disrupt and disaggregate the Aβ’s fibrils into monomers, obtaining non-toxic forms of β-secretase’s products^34^. To assess the effect of these new hybrids in the inhibition of amyloid peptide aggregation, a selection of the compounds was *in vitro* assayed, based on the thioflavin T (ThT) method^35^. Indeed ThT is a histochemical dye able to create ionic or hydrophobic interactions with the peptide β–sheets, the predominant secondary structure of the amyloid fibrils in aqueous solutions^36^. This binding interactions can be monitored by fluorimetry since the presence of ThT-fibrils increases the absorbance and the emission of the ThT dye, and induces red shifts on the absorbance (from 385 to 446 nm) and emission peaks (from 445 to 485 nm)^14^. All the measurements were performed after incubation of the self-mediated and Cu^2+^-induced Aβ aggregates in the presence/absence of the herein studied compounds. The results, expressed as percentage of aggregation inhibition, are summarised in Table 2 and graphically presented in Figure 5(**D**), including the values obtained for Tacrine (20% of inhibition) as standard reference for comparison purpose. They showed that all compounds can inhibit the Aβ aggregation with good-to-moderate activity and improved as compared with TAC. Their activity and their dependence on ligand structural features may be rationalised by different intercalation into the fibrils, as already postulated and reported for other inhibitors^14^^,^^37^. Among the compounds in study, **20** exhibited the highest inhibitory activity in both kinds of induced Aβ aggregation, while others (ex. **17**, **18**, **19**) present also very good activity for self-mediated Aβ aggregation, but they are expected to have moderate activity for Cu^2+^-induced Aβ aggregation due to absence of the OH group. Interestingly, the hydroxyl substituent (**21**–**24)** seems to lead to a general inhibitory activity decrease of the self-mediated Aβ aggregation (as compared with the non-substituted or the metoxy-substituted analogues), while the inhibitory capacity towards the Cu^2+^-induced Aβ aggregation increased, as compared with conditions of metal absence. This may be rationalised on the basis of their copper chelating capacity, as previously shown by other metal chelating compounds^14^^,^^38^. Furthermore, despite some exceptions (e.g. **20** and **24**), the increase in linker length and the chloro TAC substitution induce some small negative effects in the inhibition of Aβ self-aggregation.

**Figure 6.**
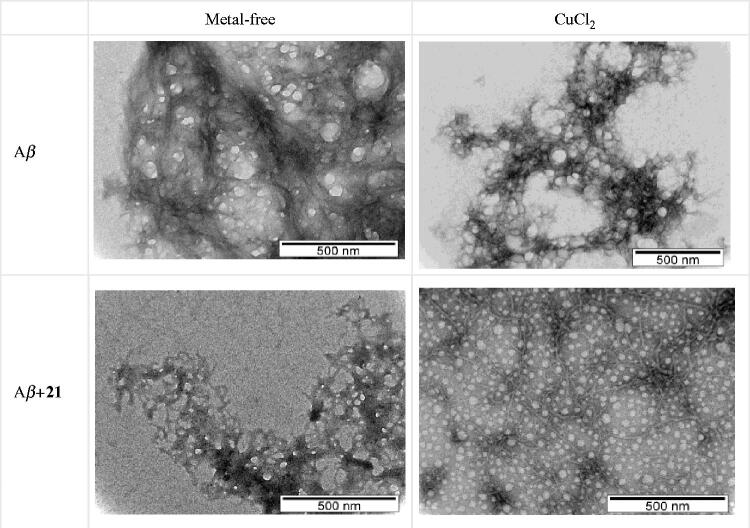
TEM images of Aβ aggregation inhibition performed with samples incubated (37 °C) for 24 h. Experimental conditions: [A*β*_1–42_] = [CuCl_2_] = 25 μM; [**21**] = 50 μM; pH = 6.6.

Nevertheless, since quantitative analysis based on fluorescence of copper(II) containing solutions may result in somehow erroneous conclusions due to some potential emission quenching by this paramagnetic ion, a fluorescence-independent study by transmission electron microscopy (TEM) was also performed for compound **21**. The TEM images depicted in Figure 6 show the presence of heavily intertwined aggregates, for Aβ_42_ alone, while in the case of its co-incubation with **21** the aggregates became sparser and more elongated, thus suggesting that for this TAC-BF hybrid the Aβ self-induced aggregation maybe be mainly attributed to the ligand intercalation between β-sheets of Aβ fibrils. The presence of copper induced changes in the morphology of the Aβ aggregates, both in the absence and in the presence of **21**, although in this last case there is a considerable change, apparently with the formation of homogenous low molecular aggregates. Overall, TEM confirms the role of the ligand in the inhibition of Aβ aggregation.

#### 2.4.3. Pharmacokinetic parameters

To assess the drug-likeness of these new drug candidates, some relevant pharmacokinetic parameters were *in silico* calculated to identify possible peripheral or central toxicity and to evaluate their ability to cross membranes, namely the blood-brain barrier (BBB), in case of drugs which main goal is the CNS. Thus, all the studied compounds have been evaluated by QikProp V. 2.5 program^39^, to gain prediction on some pharmacokinetic parameters, such as: the lipophilic character, (the octanol–water partition coefficient, *c*log *P*), the capacity to cross the BBB (log BB), the velocity of intestinal absorption (caco-2 cell permeability), the activity in the CNS and also the verification of Lipinski’s rule of five, to give an idea of potential future oral formulation as anti-AD agents (see Table 3).

**Table 3.** Summary of calculated pharmacokinetic molecular descriptors for the new hybrids by QikProp v.2.540^39^

Comp. Ref.	(n,R,R1)	MW^a^	*c*log *P*^b^	log BB^c^	Caco-2 Permeability^d^ (nm/sec)	Violations of Lipinski's rule of 5^e^	CNS activity^f^
**13**	(1,H,H)	399.491	5.143	−0.690	1764	1	+/−
**14**	(2,H,H)	413.518	5.106	−0.488	2173	1	+/−
**15**	(1,Cl,H)	433.936	5.685	−0.396	2149	1	+/−
**16**	(2,Cl,H)	447.963	6.039	−0.559	1893	1	+/−
**17**	(1,H,OCH_3_)	429.518	5.327	−0.573	2423	1	+/−
**18**	(2,H,OCH_3_)	443.544	5.745	−0.802	1867	1	–
**19**	(1,Cl,OCH_3_)	463.963	5.852	−0.431	2324	1	+/−
**20**	(2,Cl,OCH_3_)	477.989	5.907	−0.597	1890	1	+/−
**21**	(1,H,OH)	415.491	4.567	−1.522	460	0	–
**22**	(2,Cl,OH)	429.518	4.755	−1.466	520	0	–
**23**	(1,Cl,OH)	449.936	4.764	−1.261	469	0	–
**24**	(2,Cl,OH)	463.963	5.208	−1.324	493	1	–

a MW < 500.

b −2 < *c*log P < −6.5.

c −3.0 < log BB < 1.2.

d <25 poor and >500 great.

e Maximum is 4.

f (–) inactive toxicologically – (++) very active toxicologically^39^.

Analysis of the results depicted in Table 3 shows that important parameters, such as molecular weight, *c*log *P* and log BB, are perfectly inside the range determined by Lipinski’s rule to act in the CNS, though it has been noticed that one parameter has been violated except for **21**, **22** and **23**. In fact, most of the values calculated for *clog P* are close to the limit imposed by the used range, warning for possible negative consequences on the absorption through the BBB due to their lipophilic components (presence of longer chain or absence of polar hydroxyl groups). Furthermore, all the compounds evidenced good capacity to cross the intestinal cell barrier for a good GUT absorption, though the last four compounds have lower values, apparently due to the presence of that hydrophilic group. Lastly, but not less important, the predicted CNS activity, related to toxicological effects, indicates they should be safe and without anticipated side effects.

#### 2.4.4. Cell viability and neuroprotection

**Figure 7.**
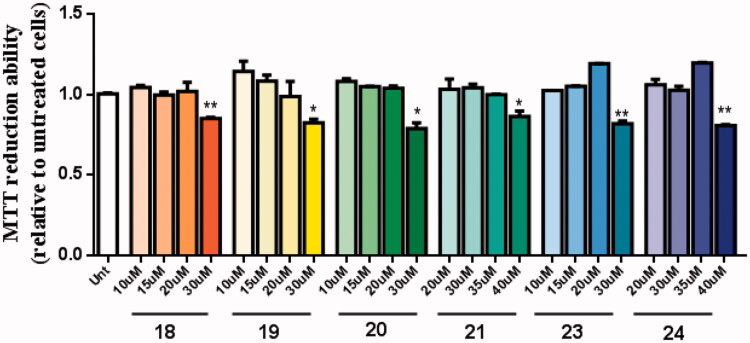
Dose-response screening to select non-toxic concentrations of TAC-BF hybrids. Cells were treated with varying concentration of TAC-BF conjugates (from 10 μM to 40 μM) for 25 h and cell viability was determined using the MTT reduction assay. Results are expressed relatively to SH-SY5Y untreated cells, with the mean ± SEM derived from three different experiments. *p < 0.05; **p < 0.01, significantly different when compared with SH-SY5Y untreated cells.

The neuroprotective effect of these new multi-target drugs designed for AD was evaluated using SH-SY5Y cells treated with Aβ_1-42_ peptides or ascorbate/iron. For each compound we performed a dose-response curve to select a non-toxic concentration (Figure 7).

**Figure 8.**
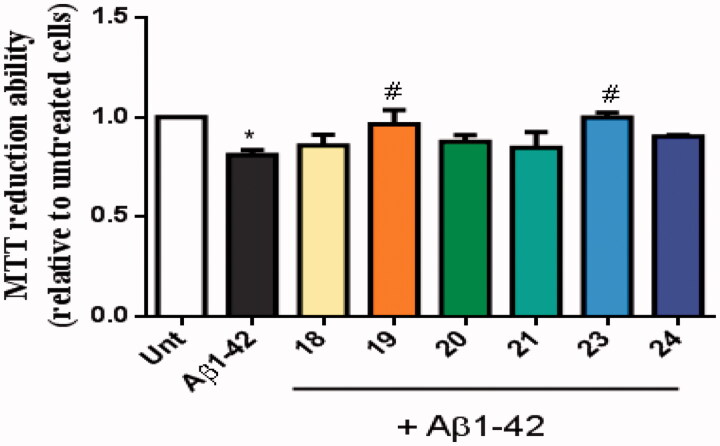
Neuroprotective effect of TAC-BF hybrids from Aβ_42_-induced toxicity on SH-SY5Y cells. Cells were treated with Aβ_42_ peptide (2.5 μM) for 24 h in the absence or in the presence of the compounds (1 h pre-incubation + 24 h co-incubation). Evaluation of cell viability was performed using the MTT reduction assay. Results are expressed relatively to SH-SY5Y untreated cells, with the mean ± SEM derived from 4 different experiments. *p < 0.05, significantly different when compared with SH-SY5Y untreated cells; #*p* < 0.05, significantly different when compared with Aβ_42_ treated SH-SY5Y cells. (compounds: **18**, **19**, **20** and **23** – 20 µM; **21**, **24** – 35 µM).

AD is characterised by the extracellular accumulation of senile plaques composed of aggregated amyloid-beta peptide (Aβ). Aβ peptides are synthesised inside cells where they oligomerize and may react with active metal-ions such as copper, iron or zinc. The production of ROS conjugated with oligomeric Aβ peptide is involved in the neurodegenerative process of AD^40^. We observed that Aβ_42_ decreased cellular viability and, interestingly, the TAC–BF conjugates **19** and **23** prevented that Aβ-induced cell toxicity (Figure 8).

**Figure 9.**
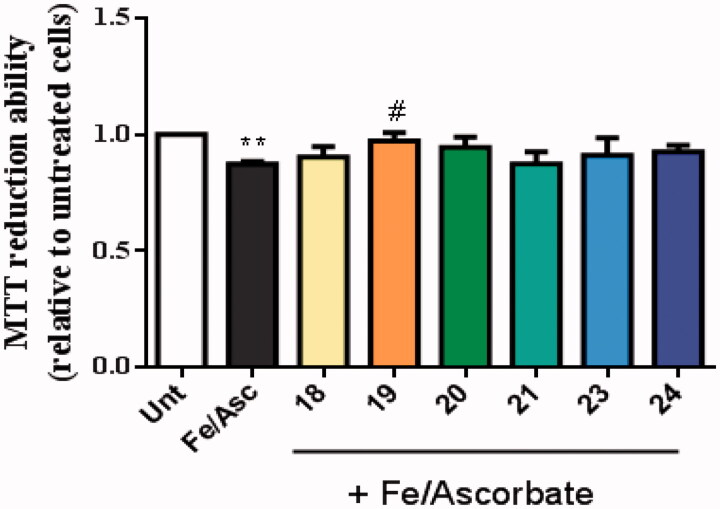
Neuroprotective effect of TAC-BF conjugates against L-Ascorbic Acid (AscH(-))/Ferrous Sulphate (Fe) toxicity on SH-SY5Y cells. Cells were treated with Asc/Fe (5 mM/500 μM, respectively) for 24 h, after treatment for 1 h in the absence or in the presence of the compounds. Evaluation of cell viability was performed by using MTT reduction assay. Results are expressed relatively to SH-SY5Y untreated cells, with the mean ± SEM derived from 4 different experiments. **p < 0.01, significantly different when compared with SH-SY5Y untreated cells. #*p* < 0.05, significantly different when compared with Asc/Fe treated SH-SY5Y cells. (compounds: **18**, **19**, **20** and **23** – 20 µM; **21**, **24** – 35 µM).

Compound **19** showed ability to inhibit Aβ-self-aggregation, thus being expected to decrease the formation of oligomers and ROS-dependent production. In addition, oxidative stress is an early event in the course of AD neurodegenerative process and it is associated with increased oxidation of proteins, lipids and nucleic acids in AD hippocampus and cortex. These data correlates with the elevated levels of Aβ_40_ and Aβ_42_ in the same brain regions^40^. To induce oxidative stress in our cellular model we used the pair Asc/Fe, which showed a decrease in the viability of SHSY-5Y cells (Figure 9). Nevertheless, among the new TAC-BF hybrids tested, compound **19** appeared to best achieve cell protection from Asc/Fe-induce oxidative stress, thus presenting the best radical scavenging properties in cell environment. Therefore, although in solution conditions the hydroxyl-containing compounds (**21**–**24**) revealed higher radical scavenging capacity than the tested non-hydroxyl compound (**20**), that feature was not paralleled in cell environment. This may be due to the higher hydrophilic character of these compounds (see Table 3). In fact, compound **21** evidenced the worst values calculated for BBB and Caco permeability and presented also the lowest capacity for in cell radical scavenging.

## 3. Conclusion

A new set of tacrine-benzofuran hybrids was designed, synthesised and studied through a multidisciplinary approach. These new compounds evidenced very good capacity for inhibition of AChE (submicromolar range), with the best activity found for compounds **15, 19, 23**, with IC_50_ values much lower than that of tacrine (TAC). Therefore, according to the design aims, the extra-functional benzofuran (BF) moiety enabled the hybrids with dual-binding capacity in their interaction with the active binding sites (CAS and PAS) of the enzyme. Structure activity relationships, indicate that the best activity corresponds to compounds with a shorter linker (propylic chain) and chloride substituent at C6 position of TAC; the substituents at the C7 of BF did not evidence considerably systematic effects in the inhibitory activity, but the –OH causes a general small decrease. Regarding the inhibition of Aβ aggregation, the activity seems to be mainly related to the intercalating ability of the compound inside the fibrils. The best capacity for inhibition of Aβ self-aggregation was presented by **20** followed by compounds **17**, **18**, **19**. The OH-containing compounds present a general decrease in the self-aggregation inhibition although, in the presence of Cu^2+^, the inhibition is generally increased attributed to metal chelating effect. Furthermore compounds **19** and **23** presented the best achievements in terms of cell neuroprotective effects. Overall, a set of TAC-BF hybrids presented very good properties for potential drugs against AD and they deserve to be further explored for the challenging discovery of new AD therapies.

## 4. Experimental part

### 4.1. Materials and equipment

Analytical grade reagents were purchased from Sigma-Aldrich and Acros, and were used as supplied. Solvents were dried according to standard methods^41^. The chemical reactions were monitored by Thin Layer Chromatography (TLC) using alumina plates coated with silica gel 60 F254 (Macherey-Nagel). Column chromatography purifications were performed on silica gel (Merck 230–400 mesh (Geduran Si 60). The melting points (mp) were measured with a Leica Galen III hot stage apparatus and are uncorrected. The Proton and Carbon-13 NMR were recorded either on Bruker AVANCE III-300 (300 MHz and 75.5 MHz) or Bruker AVANCE III-400 (400 MHz and 100.5 MHz) NMR spectrometers, at 25 °C. Chemical shifts (δ) are reported in ppm from the standard internal reference tetramethylsilane (TMS) and the coupling constant values (*J*) are determined in Hertz. In ^1^H NMR description, the following abbreviations are used: s = singlet, d = doublet, t = triplet, m = multiplet, *dt* = double triplet, *dd* = double doublet, *td* = triple doublet, *bs* = broad singlet, *bd* = broad doublet. Mass spectra (ESI-MS) were performed on a 500 MS LC Ion Trap mass spectrometer (Varian Inc., Palo Alto, CA, USA) equipped with an ESI ion source, operated in the positive ion mode. High-resolution mass spectra (HRMS) were obtained on a Bruker Impact II quadrupole mass spectrometer (Bruker Daltoniks).

For the metal complexation studies, the aqueous iron (FeCl_3_, 0.0177 M) and copper (CuCl_2_, 0.015 M) stock solutions were prepared from 1000 ppm standards (Titrisol) and their metal content was evaluated by atomic absorption. To prevent hydrolysis, the iron stock solution was prepared in excess of acid chloride and its exact concentration in HCl determined by the usual standard-addition method using 0.1 M HCl (Titrisol). The 0.1 M HCl solution, used in spectrophotometric titrations, was prepared from a Titrisol ampoule while the titrant was from carbonate free commercial concentrate (Titrisol, KOH 0.1 M ampoules). The KOH solution was standardised by titration with a solution of potassium hydrogen phthalate and was discarded whenever the percentage of carbonate, determined by Gran’s method^42^, was greater than 0.5% of the total amount of base.

The electronic spectra and the AChE inhibition assays were performed in a 1-cm path length quartz cell with a Perkin Elmer Lambda 35 spectrophotometer equipped with a temperature programmer PTP1 + 1 Peltier System (T = 25.0 ± 0.1 °C). The enzyme AChE 500 U, extracted from *Electrophorus electricus* (electric eel), was purchased from Sigma-Aldrich.

For the study of the β-amyloid (Aβ) aggregation, the ThT fluorescence assay was performed using a Varian Cary Eclipse fluorimeter at the following wavelengths: excitation (446 nm) and emission (485 nm). Amyloid *β*-peptide (1–42), A*β*_1-42_, was purchased from GeneCust as a lyophilised powder stored at −20 °C. TEM assays were performed with a Hitachi H8100 transmission electron microscope with a LaB6 filament (200 kV, 10000–20000 × magnification) at MicroLab/IST.

### 4.2. Synthesis of the compounds

The schematic representation and the synthetic steps involved in the preparation of all TAC-BF hybrids and their intermediates are shown in Scheme 1.

#### 4.2.1. General procedure for synthesis of 9-chloro-1,2,3,4-tetrahydroacridine (3 − 4)

The title compounds, 9-chloro-1,2,3,4-tetrahydroacridine (**3**) and 6,9-dichloro-1,2,3,4-tetrahydroacridine (**4**), were prepared from commercially available anthranilic acids according to our previous reported procedure^14^^,^^16^.

#### 4.2.2. General procedure for synthesis of ethyl benzofuran-2-carboxylate (7 − 8)

To a solution of substituted salicyaldehydes (72 mmol) and potassium carbonate (10 g, 72 mmol) in DMF (100 mL) was added ethylbromoacetate (12 g, 72 mmol). The reaction was refluxed in oil bath for 12 h and after completion of reaction, it was poured in cold water and the product was extracted with ethyl acetate^19^. The crude product was purified by column chromatography using 1% ethyl acetate-petroleum ether as eluent to yield the desired product (70 − 75%).

#### 4.2.3. Ethyl benzofuran-2-carboxylate (7)

Yield 70%, mp 48 − 50 °C; ^1^H NMR (CDCl_3_, 300 MHz) *δ*: 7.69 (d, 1H, *J* = 2.0 & 7.8 Hz, H-4), 7.60 (d, 1H, *J* = 8.4 Hz, H-7), 7.54 (s, 1H, H-3), 7.43–7.48 (m, 1H, H-6), 7.26–7.33 (m, 1H, H-5), 4.45 (q, 2H, *J* = 7.2 Hz, *CH_2_*CH_3_), 1.44 (t, 3H, *J* = 6.9 Hz, *CH_3_*CH_2_); ^13^C NMR (CDCl_3_, 100 MHz) *δ*: 159.59, 155.67, 145.69, 127.53, 126.94, 123.73, 122.76, 113.76, 112.35, 61.50, 14.32; *m/z* (ESI-MS): 190.54 [M]^+^.

#### 4.2.4. Ethyl 7-methoxybenzofuran-2-carboxylate (8)

Yield 75%, mp 56 − 57 °C; ^1^H NMR (CDCl_3_, 300 MHz) *δ*: 7.51 (s, 1H, H-3), 7.18 − 7.25 (m, 2H, H-4 and H-5), 7.10 (d, 1H, *J* = 7.8 Hz, H-6), 4.34 (q, 2H, *J* = 7.2 Hz, *CH_2_*CH_3_), 3.94 (s, 3H, OCH_3_), 1.32 (t, 3H, *J* = 7.2 Hz, *CH_3_*CH_2_); ^13^C NMR (CDCl_3_, 100 MHz) *δ*: 159.34, 145.95, 145.93, 145.35, 128.57, 124.37, 114.52, 114.00, 108.86, 61.38, 56.00, 14.30; *m/z* (ESI-MS): 243.08 [M + Na]^+^.

#### 4.2.5. General procedure for synthesis of N-(aminoalkyl)benzofuran-2-carboxamide (9–12)

To the mixture of diaminoalkane (4 eq) in methanol, 1 M solution of ethyl benzofuran-2-carboxylate (**7**, **8**) in methanol was added dropwise over a period of 5 h at room temperature. The reaction mixture was stirred overnight and completion of reaction was monitored on TLC. On completion, the reaction mixture was concentrated under reduced pressure and the residue so obtained was diluted 4 M HCl solution (100 mL) under ice cold condition and washed with DCM (3 × 25 mL). Finally the aqueous layer was basified with saturated sodium hydroxide solution to 11–12 pH, extracted with ethyl acetate and dried on sodium sulphate. The evaporation of ethyl acetate under reduced pressure gave the desired pure *N*-(aminoalkyl)benzofuran-2-carboxamides (**9**–**12**) as light yellow solids in 64 − 71% yield.

#### 4.2.6. N-(3-aminopropyl)benzofuran-2-carboxamide (9)

The title compound was synthesised from reaction of compound **8** with 1,3-diaminopropane. Yield 64%; mp 120–121 °C. ^1^H NMR (400 MHz, MeOD-*d_4_*) *δ*: 7.64 (d, 1H, *J* = 8.0 Hz, H-4), 7.52 (d, 1H, *J* = 8.0 Hz, H-7), 7.44 (s, 1H, H-3), 7.39 (t, 1H, *J* = 8.0 Hz, H-6), 7.25 (t, 1H, *J* = 8.0 Hz, H-5), 3.48 (t, 2H, *J* = 8.0 Hz, *CH_2_*NHCO), 2.72 (t, 2H, *J* = 8.0 Hz, *CH_2_*NH_2_), 1.75–1.82 (m, 2H, CH_2_*CH_2_*CH_2_); ^13^CNMR (100 MHz, MeOD-d_4_) *δ*: 159.89, 154.93, 148.67, 127.39, 126.74, 123.47, 122.34, 111.38, 109.76, 38.42, 36.39, 32.09; *m/z* (ESI-MS): 219.26 [M + H]^+^.

#### 4.2.7. N-(4-aminobutyl)benzofuran-2-carboxamide (10)

The title compound was synthesised from reaction of compound **8** with 1,4-diaminobutane. Yield 69%; mp 94–95 °C. ^1^H NMR (400 MHz, MeOD-*d_4_*) *δ*: 7.70 (d, 1H, *J* = 8.0 Hz, H-4), 7.57 (d, 1H, *J* = 8.0 Hz, H-7), 7.42 − 7.47 (m, 2H, H-3 & H-6), 7.30 (t, 1H, *J* = 8.0 Hz, H-5), 3.44 (t, 2H, *J* = 8.0 Hz, *CH_2_*NHCO), 2.73 (t, 2H, *J* = 8.0 Hz, *CH_2_*NH_2_), 1.55–1.71 (m, 4H, CH_2_CH_2_*CH_2_*CH_2_); ^13^CNMR (100 MHz, MeOD-d_4_) *δ*: 159.81, 154.98, 148.74, 127.42, 126.73, 123.48, 122.33, 111.36, 109.70, 40.52, 38.71, 28.92, 26.48; *m/z* (ESI-MS): 233.37 [M + H]^+^.

#### 4.2.8. N-(3-aminopropyl)-7-methoxybenzofuran-2-carboxamide (11)

The title compound was synthesised from reaction of compound **8** with 1,3-diaminopropane. Yield 66%; mp 116 − 117 °C. ^1^H NMR (400 MHz, MeOD-*d_4_*) *δ*: 7.43 (s, 1H, H-3), 7.17 − 7.24 (m, 2H, H-4 & H-5), 6.96 (d, 1H, *J* = 8.0 Hz, H-6), 3.98 (s, 1H, OCH_3_) 3.50 (t, 2H, *J* = 8.0 Hz, *CH_2_*NHCO), 2.75 (t, 2H, *J* = 8.0 Hz, *CH_2_*NH_2_), 1.77 − 1.84 (m, 2H, CH_2_*CH_2_*CH_2_); ^13^CNMR (100 MHz, MeOD-*d*_4_) *δ*: 159.82, 148.60, 145.66, 144.40, 128.93, 124.24, 114.09, 110.06, 108.33, 55.10, 38.34, 36.33, 31.96; *m/z* (ESI-MS): 249.45 [M + H]^+^.

#### 4.2.9. N-(4-aminobutyl)-7-methoxybenzofuran-2-carboxamide (12)

The title compound was synthesised from reaction of compound **8** with 1,4-diaminobutane. Yield 71%; mp 89 − 90 °C. ^1^H NMR (400 MHz, MeOD-*d_4_*) *δ*: 7.44 (s, 1H, H-3), 7.18 − 7.25 (m, 2H, H-4 & H-5), 6.97 (d, 1H, *J* = 8.0 Hz, H-6), 3.98 (s, 1H, OCH_3_) 3.43 (t, 2H, *J* = 8.0 Hz, *CH_2_*NHCO), 2.70 (t, 2H, *J* = 8.0 Hz, *CH_2_*NH_2_), 1.54–1.69 (m, 2H, CH_2_*CH_2_CH_2_*CH_2_); ^13^CNMR (100 MHz, MeOD-*d_4_*) *δ*: 159.68, 148.70, 145.66, 144.39, 128.95, 124.26, 114.11, 110.03, 108.32, 55.12, 40.75, 38.82, 29.50, 26.51;; *m/z* (ESI-MS): 263.44 [M + H]^+^.

#### 4.2.10. General procedure for the synthesis of N-(4-((1,2,3,4-tetrahydroacridin-9-yl)amino)alkyl)benzofuran-2-carboxamide derivatives (13 − 20)

The mixture of 9-chloro-1,2,3,4-tetrahydroacridine (**3**, **4**) (1 eq) and *N*-(aminoalkyl)benzofuran-2-carboxamide (**9 **−** 12**) (1 eq) was heated in phenol (0.5 eq) with the catalytic amount of potassium iodide at 155 − 160 °C for 35 − 60 min. The completion of reaction was monitored on TLC. On completion, the dense oily reaction mixture was sonicated with ethyl acetate. The precipitates so obtained were filtered and washed with water and hexane to give light yellow coloured *N*-(4-((1,2,3,4-tetrahydroacridin-9-yl)amino)butyl)benzofuran-2-carboxamide derivatives (**13**–**20**); the compounds were purified either through crystallisation in ethylacetate-hexane or through column chromatography over silica (0.5 − 1.0% MeOH-DCM) in 67 − 79% yield.

#### 4.2.11. N-(3-((1,2,3,4-tetrahydroacridin-9-yl)amino)propyl)benzofuran-2-carboxamide (13)

The title compound was synthesised from reaction of compounds **3** and **9**. Yield 67%; mp 145 − 146 °C. ^1^H NMR (400 MHz, MeOD-*d_4_*) *δ*: 8.39 (d, 1H, *J* = 8.0 Hz, H-8), 7.71 − 7.77 (m, 3H, H-5, H-6 & H-4′), 7.48−.57 (m, 3H, H-7, H-7′ & H-6′′), 7.42 (s, 1H, H-3′), 7.34 − 7.36 (m, 1H, H-5′), 4.09 (brs, 2H, *CH_2_*NH), 3.59 (brs, 2H, *CH_2_*NHCO), 2.94 (brs, 2H, H-4), 2.75 (brs, 2H, H-1), 2.17 (brs, 2H, CH_2_*CH_2_*CH_2_), 1.92 (brs, 4H, H-2 & H-3); ^13^CNMR (100 MHz, MeOD-*d_4_*) *δ*: 160.16, 156.69, 154.98, 150.31, 148.32, 138.33, 132.55, 127.32, 126.94, 124.89, 123.60, 122.39, 118.64, 115.66, 111.63, 111.37, 109.92, 44.98, 36.05, 29.94, 27.87, 23.55, 21.54, 20.34; HRMS (ESI) calcd for C_25_H_26_N_3_O_2_ [M + H]^+^, *m/z* 400.2020, found 400.2034.

#### 4.2.12. N-(4-((1,2,3,4-tetrahydroacridin-9-yl)amino)butyl)benzofuran-2-carboxamide (14)

The title compound was synthesised from reaction of compounds **3** and **10**. Yield 72%; mp 117 − 118 °C. ^1^H NMR (400 MHz, MeOD-*d_4_*) *δ*: 8.30 (d, 1H, *J* = 8.0 Hz, H-8), 7.69 (t, 1H, *J* = 8.0 Hz, H-6), 7.60 − 7.63 (m, 2H, H-5 & H-4′), 7.45 − 7.49 (m, 2H, H-7 & H-7′), 7.39 (t, 1H, *J* = 8.0 Hz, H-6′), 7.32 (s, 1H, H-3′), 7.25 (t, 1H, *J* = 8.0 Hz, H-5′), 3.96 (t, 2H, *J* = 8.0 Hz, *CH_2_*NH), 3.42 (t, 2H, *J* = 8.0 Hz, *CH_2_*NHCO), 2.89 (brs, 2H, H-4), 2.62 (brs, 2H, H-1), 1.87 (brs, 6H, H-2, H-3 & NHCH_2_*CH_2_*) 1.73 − 1.77(m, 2H, *CH_2_*CH_2_CONH); ^13^CNMR (100 MHz, MeOD-d_4_) *δ*: 159.73, 156.28, 154.86, 150.30, 148.52, 138.37, 132.43, 127.29, 126.79, 125.00, 124.88, 123.52, 122.30, 118.79, 115.72, 111.60, 111.34, 109.65, 38.48, 27.98, 27.42, 25.99, 23.47, 21.57, 20.40; HRMS (ESI) calcd for C_26_H_28_N_3_O_2_ [M + H]^+^, *m/z* 414.2176, found 414.2147.

#### 4.2.13. N-(3-((6-chloro-1,2,3,4-tetrahydroacridin-9-yl)amino)propyl)benzofuran-2-carboxamide (15)

The title compound was synthesised from reaction of compounds **4** and **9**). Yield 73%; mp 124 − 125 °C. ^1^H NMR (400 MHz, MeOD-*d_4_*) *δ*: 8.17 (d, 1H, *J* = 8.0 Hz, H-8), 7.67 (d, 1H, *J* = 8.0 Hz, H-4′), 7.60 (s, 1H, H-5) 7.52 (d, 1H, *J* = 8.0 Hz, H-7′), 7.43 (t, 1H, *J* = 8.0 Hz, H-6′), 7.39 (s, 1H, H-3′), 7.27 − 7.32 (m, 2H, H-5′ & H-7), 3.86 (t, 2H, *J* = 8.0 Hz, *CH_2_*NH), 3.55 (t, 2H, *J* = 8.0 Hz, *CH_2_*NHCO), 2.87 (brs, 2H, H-4), 2.66 (brs, 2H, H-1), 2.06–2.09 (m, 2H, CH_2_*CH_2_*CH_2_) 1.85–1.86(m, 4H, H-2, H-3); ^13^CNMR (100 MHz, MeOD-d_4_) *δ*: 160.07, 154.90, 154.61, 153.49, 148.32, 141.69, 136.88, 127.26, 126.91, 126.24, 124.68, 123.56, 122.36, 120.28, 115.27, 113.20, 111.34, 109.89, 45.05, 36.15, 30.08, 29.61, 23.89, 21.78, 20.87; HRMS (ESI) calcd for C_25_H_25_ClN_3_O_2_ [M + H]^+^, *m/z* 434.6482, found 434.6449.

#### 4.2.14. N-(4-((6-chloro-1,2,3,4-tetrahydroacridin-9-yl)amino)butyl)benzofuran-2-carboxamide (16)

The title compound was synthesised from reaction of compounds **4** and **10**. Yield 70%; mp 127 − 129 °C. ^1^H NMR (400 MHz, MeOD-*d_4_*) *δ*: 8.32 (d, 1H, *J* = 8.0 Hz, H-8), 7.72 (d, 1H, *J* = 8.0 Hz, H-4′), 7.60 (s, 1H, H-5) 7.53 (d, 1H, *J* = 8.0 Hz, H-7′), 7.45 − 7.49 (m, 2H, H-6′ & H-7), 7.39 (s, 1H, H-3′), 7.34 (t, 1H, *J* = 8.0 Hz, H-5′), 3.95 (t, 2H, *J* = 8.0 Hz, *CH_2_*NH), 3.45 (t, 2H, *J* = 8.0 Hz, *CH_2_*NHCO), 2.93 (brs, 2H, H-4), 2.69 (brs, 2H, H-1), 1.90–1.93 (m, 6H, H-2, H-3 & NHCH_2_*CH_2_*) 1.77–1.81(m, 2H, *CH_2_*CH_2_CONH); ^13^CNMR (100 MHz, MeOD-*d_4_*) *δ*: 159.75, 155.41, 154.91, 152.37, 148.52, 140.66, 137.71, 127.32, 126.84, 125.03, 123.56, 122.35, 119.21, 114.92, 112.88, 111.31, 109.67, 38.47, 28.93, 27.32, 25.87, 23.52, 21.66, 20.67; HRMS (ESI) calcd for C_26_H_27_ClN_3_O_2_ [M + H]^+^, *m/z* 448.6636, found 448.6677.

#### 4.2.15. 7-Methoxy-N-(3-((1,2,3,4-tetrahydroacridin-9-yl)amino)propyl)benzofuran-2-carboxamide (17)

The title compound was synthesised from the reaction of compounds **3** and **11**. Yield 75%; mp 137 − 138 °C. ^1^H NMR (400 MHz, MeOD-d_4_) *δ*: 8.28 (d, 1H, *J* = 8.0 Hz, H-8), 7.62 − 7.70 (m, 2H, H-5 & H-6), 7.45 (t, 1H, *J* = 8.0 Hz, H-7), 7.33 (s, 1H, H-3′) 7.19 − 7.20 (m, 2H, H-4′ & H-5′), 6.96 − 6.97 (m, 1H, H-6′), 3.98 (t, 2H, *J* = 8.0 Hz, *CH_2_*NH), 3.94 (s, 3H, OCH_3_), 3.52–3.53 (m, 2H, *CH_2_*NHCO), 2.85 (brs, 2H, H-4), 2.66 (brs, 2H, H-1), 2.08–2.13 (m, 2H, NHCH_2_*CH_2_*) 1.84 (brs, 4H, H-2 & H-3); ^13^C NMR (100 MHz, MeOD-*d_4_*) *δ*: 159.98, 156.02, 150.85, 148.32, 145.67, 144.40, 138.99, 132.08, 128.84, 124.72, 124.37, 119.34, 115.94, 114.08, 111.84, 110.17, 108.43, 55.07, 45.00, 36.15, 29.95, 28.27, 23.64, 21.60, 20.47; HRMS (ESI) calcd for C_26_H_28_N_3_O_3_ [M + H]^+^, *m/z* 430.2135, found 430.2142.

#### 4.2.16. 7-Methoxy-N-(4-((1,2,3,4-tetrahydroacridin-9-yl)amino)butyl)benzofuran-2-carboxamide (18)

The title compound was synthesised from the reaction compounds **3** and **12**. Yield 79%; mp 111 − 113 °C. ^1^H NMR (400 MHz, MeOD-*d_4_*) *δ*: 8.23 (d, 1H, *J* = 8.0 Hz, H-8), 7.62 − 7.68 (m, 2H, H-5 & H-6), 7.43 (t, 1H, *J* = 8.0 Hz, H-7), 7.37 (s, 1H, H-3′) 7.24 − 7.25 (m, 2H, H-4′ & H-5′), 7.02 (d, 1H, *J* = 8.0 Hz, H-6′), 3.99 (s, 3H, OCH_3_), 3.80 (t, 2H, *J* = 8.0 Hz, *CH_2_*NH), 3.43 (t, 2H, *J* = 8.0 Hz, *CH_2_*NHCO), 2.93 (brs, 2H, H-4), 2.71 (brs, 2H, H-1), 1.90 (brs, 4H, H-2 & H-3), 1.73–1.86 (m, 4H, NHCH_2_*CH_2_CH_2_*); ^13^C NMR (100 MHz, MeOD-*d_4_*) *δ*: 159.66, 154.03, 148.57, 145.71, 144.42, 142.40, 130.36, 128.93, 124.30, 124.12, 123.95, 117.86, 114.09, 113.62, 109.99, 108.37, 55.05, 38.53, 30.33, 27.77, 26.15, 24.06, 22.10, 21.30; HRMS (ESI) calcd for C_27_H_30_N_3_O_3_^+^ [M + H]^+^, *m/z* 444.2282, found 444.2260.

#### 4.2.17. N-(3-((6-chloro-1,2,3,4-tetrahydroacridin-9-yl)amino)propyl)-7-methoxybenzofuran-2-carboxamide (19)

The title compound was synthesised from the reaction of compounds **4** and **11**. Yield 70%; mp 157 − 158 °C. ^1^H NMR (400 MHz, MeOD-*d_4_*) *δ*: 8.35 (d, 1H, *J* = 8.0 Hz, H-8), 7.62 (s, 1H, H-5), 7.48 (d, 1H, *J* = 8.0 Hz, H-4′), 7.35 (s, 1H, H-3′), 7.27 (brs, 2H, H-7 & H-5′), 7.05 (m, 1H, H-6′), 4.08 (brs, 2H, *CH_2_*NH), 4.01 (s, 3H, OCH_3_), 3.58 (brs, 2H, *CH_2_*NHCO), 2.87 (brs, 2H, H-4), 2.71 (brs, 2H, H-1), 2.17 (m, 2H, NHCH_2_*CH_2_*) 1.90 (brs, 4H, H-2 & H-3); ^13^CNMR (100 MHz, MeOD-*d_4_*) *δ*: 159.87, 156.56, 150.68, 148.24, 145.68, 144.39, 139.00, 138.55, 128.81, 127.11, 125.28, 124.44, 117.61, 114.11, 113.99, 112.09, 110.18, 108.52, 55.06, 45.18, 36.02, 29.52, 27.84, 23.40, 21.38, 20.22; HRMS (ESI) calcd for C_26_H_27_ClN_3_O_3_ [M + H]^+^, *m/z* 464.6577, found 464.6565.

#### 4.2.18. N-(4-((6-chloro-1,2,3,4-tetrahydroacridin-9-yl)amino)butyl)-7-methoxybenzofuran-2-carboxamide (20)

The title compound was synthesised from the reaction of compounds **4** and **12**. Yield 79%; mp 245 − 247 °C. ^1^H NMR (400 MHz, MeOD-*d_4_*) *δ*: 8.39 (d, 1H, *J* = 8.0 Hz, H-8), 7.51 − 7.52 (m, 2H, H-5 & H-4′), 7.33 (s, 1H, H-3′), 7.28 − 7.29 (m, 2H, H-7 & H-5′), 7.05 − 7.07 (m, 1H, H-6′), 4.05 (t, 2H, *J* = 8.0 Hz, *CH_2_*NH), 4.01 (s, 3H, OCH_3_), 3.45 (t, 2H, *J* = 8.0 Hz, *CH_2_*NHCO), 2.91 (brs, 2H, H-4), 2.69 (brs, 2H, H-1), 1.96–1.97 (m, 6H, NHCH_2_*CH_2_*, H-2 & H-3), (m, 2H, *CH_2_*CH_2_NHCO); ^13^CNMR (100 MHz, MeOD-*d_4_*) *δ*: 159.55, 156.17, 150.66, 148.45, 145.66, 144.32, 140.16, 138.43, 128.84, 127.27, 125.30, 124.40, 117.85, 114.08, 112.39, 109.93, 108.42, 55.04, 38.49, 28.11, 27.09, 25.45, 23.24, 21.45, 20.35; HRMS (ESI) calcd for C_27_H_29_ClN_3_O_3_ [M + H]^+^, *m/z* 478.6745, found 478.6734.

#### 4.2.19. General procedure for the deprotection of the hydroxy group (21–24)

A mixture of 7-methoxy-*N*-(4-((1,2,3,4-tetrahydroacridin-9-yl)amino)alkyl)-benzofuran-2-carboxamide (1 eq.) and *n*-Bu_4_NI (3 eq.) in dry CH_2_Cl_2_ (15 mL) was stirred under N_2_ atmosphere and cooled to −78 °C. To this solution was dropwise added an excess of a solution 1 M BCl_3_ in CH_2_Cl_2_ (5 eq.). After ca 5 min, the solution could warm to room temperature and was stirred for 2 h. The reaction was quenched with ice water and it was left to stir for 30 min. A precipitate was formed and the product was recrystallized from CH_3_CN/Et_2_O to afford the pure final compounds as a pale yellow solids. Yield: 40 − 66%.

#### 4.2.20. 7-Hydroxy-N-(3-((1,2,3,4-tetrahydroacridin-9-yl)amino)propyl)benzofuran-2-carboxamide (21)

The title compound was synthesised from compound **17**. Yield 40%; mp 93 − 95 °C. ^1^H NMR (400 MHz, MeOD-*d_4_*) *δ*: 8.28 (d, 1H, *J* = 8.0 Hz, H-8), 7.62 − 7.70 (m, 2H, H-5 & H-6), 7.45 (t, 1H, *J* = 8.0 Hz, H-7), 7.33 (s, 1H, H-3′) 7.19–7.20 (m, 2H, H-4′ & H-5′), 6.96–6.97 (m, 1H, H-6′), 3.98 (t, 2H, *J* = 8.0 Hz, *CH_2_*NH), 3.52–3.53 (m, 2H, *CH_2_*NHCO), 2.85 (brs, 2H, H-4), 2.66 (brs, 2H, H-1), 2.08–2.13 (m, 2H, NHCH_2_*CH_2_*) 1.84 (brs, 4H, H-2 & H-3); ^13^C NMR (100 MHz, MeOD-*d_4_*) *δ*: 159.98, 156.02, 150.85, 148.32, 145.67, 144.40, 138.99, 132.08, 128.84, 124.72, 124.37, 119.34, 115.94, 114.08, 111.84, 110.17, 55.07, 45.00, 36.15, 29.95, 28.27, 23.64, 21.60, 20.47. HRMS (ESI) calcd for C_25_H_25_N_3_O_3_^+^[M + H]^+^, *m/z* 415.1904, found 415.1951.

#### 4.2.21. 7-Hydroxy-N-(4-((1,2,3,4-tetrahydroacridin-9-yl)amino)butyl)benzofuran-2-carboxamide (22)

The title compound was synthesised from **18**. Yield 50%; mp 79 − 81 °C. ^1^H NMR (400 MHz, MeOD-*d_4_*) *δ*: 8.23 (d, 1H, *J* = 8.0 Hz, H-8), 7.62–7.68 (m, 2H, H-5 & H-6), 7.43 (t, 1H, *J* = 8.0 Hz, H-7), 7.37 (s, 1H, H-3′), 7.24–7.25 (m, 2H, H-4′ & H-5′), 7.02 (d, 1H, *J* = 8.0 Hz, H-6′), 3.80 (t, 2H, *J* = 8.0 Hz, *CH_2_*NH), 3.43 (t, 2H, *J* = 8.0 Hz, *CH_2_*NHCO), 2.93 (brs, 2H, H-4), 2.71 (brs, 2H, H-1), 1.90 (brs, 4H, H-2 & H-3), 1.73–1.86 (m, 4H, NHCH_2_*CH_2_CH_2_*); ^13^C NMR (100 MHz, MeOD-*d_4_*) *δ*: 159.66, 154.03, 148.57, 145.71, 144.42, 142.40, 130.36, 128.93, 124.30, 124.12, 123.95, 117.86, 114.09, 113.62, 109.99, 55.05, 38.53, 30.33, 27.77, 26.15, 24.06, 22.10, 21.30. HRMS (ESI) calcd for C_26_H_27_N_3_O_3_ [M + H]^+^, *m/z* 429.2058, found 429.2083.

#### 4.2.22. N-(3-((6-chloro-1,2,3,4-tetrahydroacridin-9-yl)amino)propyl)-7-hydroxybenzofuran-2-carboxamide (23)

The title compound was synthesised from compound **19**. Yield 50%; mp 85 − 87 °C. ^1^H NMR (400 MHz, MeOD-*d_4_*) *δ*: 8.35 (d, 1H, *J* = 8.0 Hz, H-8), 7.62 (s, 1H, H-5), 7.48 (d, 1H, *J* = 8.0 Hz, H-4′), 7.35 (s, 1H, H-3′), 7.27 (brs, 2H, H-7 & H-5′), 7.05 (m, 1H, H-6′), 4.08 (brs, 2H, *CH_2_*NH), 4.01 (s, 3H, OCH_3_), 3.58 (brs, 2H, *CH_2_*NHCO), 2.87 (brs, 2H, H-4), 2.71 (brs, 2H, H-1), 2.17 (m, 2H, NHCH_2_*CH_2_*) 1.90 (brs, 4H, H-2 & H-3); ^13^C NMR (100 MHz, MeOD-*d_4_*) *δ*: 159.87, 156.56, 150.68, 148.24, 145.68, 144.39, 139.00, 138.55, 128.81, 127.11, 125.28, 124.44, 117.61, 114.11, 113.99, 112.09, 110.18, 55.06, 45.18, 36.02, 29.52, 27.84, 23.40, 21.38, 20.22. HRMS (ESI) calcd for C_25_H_25_ClN_3_O_3_ [M + H]^+^, *m/z* 450.6442, found 450.6450.

#### 4.2.23. N-(4-((6-chloro-1,2,3,4-tetrahydroacridin-9-yl)amino)butyl)-7-hydroxybenzofuran-2-carboxamide (24)

The title compound was synthesised from compound **20**. Yield 66%; mp 100 − 101 °C; ^1^H NMR (400 MHz, MeOD-*d_4_*) *δ*: 8.39 (d, 1H, *J* = 8.0 Hz, H-8), 7.51 − 7.52 (m, 2H, H-5 & H-4′), 7.33 (s, 1H, H-3′), 7.28 − 7.29 (m, 2H, H-7 & H-5′), 7.05 − 7.07 (m, 1H, H-6′), 4.05 (t, 2H, *J* = 8.0 Hz, *CH_2_*NH), 3.45 (t, 2H, *J* = 8.0 Hz, *CH_2_*NHCO), 2.91 (brs, 2H, H-4), 2.69 (brs, 2H, H-1), 1.96–1.97 (m, 6H, NHCH_2_*CH_2_*, H-2 & H-3), (m, 2H, *CH_2_*CH_2_NHCO); ^13^C NMR (100 MHz, MeOD-*d_4_*) *δ*: 159.55, 156.17, 150.66, 148.45, 145.66, 144.32, 140.16, 138.43, 128.84, 127.27, 125.30, 124.40, 117.85, 114.08, 112.39, 109.93, 55.04, 38.49, 28.11, 27.09, 25.45, 23.24, 21.45, 20.35. HRMS (ESI) calcd for C_26_H_26_ClN_3_O_3_ [M + H]^+^, *m/z*, 464.6511, found 463.6498.

### 4.3. Molecular modelling

The interactions between ligands and proteins are of fundamental importance in the modern structure–based drug design, and so docking simulations with GOLD v. 5.1 program^43^ were performed. The inhibitor models were docked inside the protein model of *Torpedo californica* AChE (*Tc*AChE), which was obtained from the X-ray structure of its complex with an AChE inhibitor (*N*-4’-quinolyl-*N*’-9’’-(1’’,2’’,3’’,4’’-tetrahydroacridinyl)-1,8-diaminooctane, herein called original ligand), retrieved from the RCSB Protein Data Bank (PDB entry 1ODC)^20^. This structure was chosen due to the similarity between this ligand and the herein studied ligands, namely the TAC and the aromatic moiety linked by an alkylic chain, interacting respectively with the catalytic active site (CAS) and the peripheral anionic site (PAS). Structures from Homo sapiens were not selected because they are complexed with smaller inhibitors. To get the protein model, the original complex model was treated with the programme MAESTRO v. 9.3^44^ by removal of the original ligand, solvent and co-crystalized molecules, as well as by addition of hydrogen atoms. This programme was also used to design the ligand structures, which afterwards were optimised with software GHEMICAL v. 2.0^45^, through a random conformational search of 100 cycles and 2500 optimisation steps. To complete these simulations, the ligand models were docked inside the active site of the AChE structure through the programme GOLD v. 5.1, using its standard parameters and the Astex Statistical Potential (ASP) scoring function. The zone of interest of the docking was defined as the residues within 10 Å from the original position of the ligand in the crystal structure. The docking protocol used herein was validated, trough the re-docking of the co-crystallized ligand (original ligand)^20^ into the respective AChE model, leading to a structure with very good superimposition to that of reported X-ray crystal structure, as recently reported.^16^

#### 4.3.1. Prediction of pharmacokinetic properties

To get an insight on drug-likeness properties of these new hybrids, *in silico* calculations of some pharmacokinetic descriptors were performed by the QikProp program^39^ provided by MAESTRO. Parameters such as the lipo-hydrophilic character (*c*log *P*), blood-brain barrier partition coefficient (log BB), ability to be absorbed through the intestinal tract (Caco-2 cell permeability) and CNS activity were calculated. These predictions are for orally delivered drugs and assume non active transport.

### 4.4. Physico-chemical properties

#### 4.4.1. Anti-oxidant activity

The anti-oxidant capacity or, more precisely, radical scavenging activity based on the electron and proton donation ability, was evaluated by the DPPH method previously described^29^^,^^46^. To a 2.5 mL solution of DPPH (0.002%) in methanol, solutions of each compound were added in different volumes to obtain different concentrations in a 3.5 mL final volume. The samples were incubated for 30 min at room temperature. The absorbance was measured at 517 nm against the corresponding blank (methanol). The antioxidant activity was calculated by Eq. (1)^29^.

(1) $$\%\text{AA}\;=\;((A_{\text{DPPH}}-A_{\text{sample}})/A_{\text{DPPH}})\;\times \;100$$

The tests were carried out in triplicate. The compound concentration providing 50% of antioxidant activity (EC_50_) was obtained by plotting the antioxidant activity against the compound concentration.

#### 4.4.2. Metal chelation studies

### 4.5. Acetylcholinesterase inhibition

The enzymatic activity of AChE was determined using an adaptation of the Ellman’s method, as described previously^29^^,^^32^. Firstly, a stock solution of AChE was prepared by dissolving enzyme 500 U in 10 mL of tris(hydroxymethyl)aminomethane (TRIS) buffer (50 mM, pH 8). Then, 4–(2-hydroxyethyl)-1-piperazineethanesulfonic acid (HEPES) buffer was used for further dilution of the enzyme solution therefore obtaining the final working solution of AChE. The assay solution consisted of 374 µL of HEPES buffer (50 mM and pH 8.0), a variable volume (10–50 µL) of the compound’s stock solution (1 mg/mL of MeOH), 476 µL of 3 mM bis(3-carboxy-4-nitrophenyl) disulphide (DTNB), 25 µL of AChE stock solution and the necessary amount of methanol to attain the same volume of sample mixture in a 1 mL cuvette. After mixing, samples were left to incubate for 15 min and then 75 µL of 16 mM acetylthiocholine iodide (AChI) solution was added and immediately the reaction was monitored at 405 nm for 5 min. Firstly, enzyme activity was determined by measuring the rate of enzymatic reaction in solution by recording absorbance (with or without enzyme) at 405 nm. To calculate the percent inhibition of AChE, the enzyme activity in the presence of increasing concentrations of the test compound, as well as for a blank reaction (methanol without compounds), were measured at 405 nm.

The obtained results were expressed in percentage of inhibition through the Equation (2):

(2) $$\%1=100-(v_{1}/v_{0}\times 100)$$

Where *v_1_*is the initial reaction rate in the presence of the inhibitor and *v*_0_ is the initial rate of the control reaction. The curves of inhibition were obtained using a plot of percentage of enzymatic inhibition versus inhibitor concentration, and a calibration curve was obtained from which the linear regression parameters were obtained.

### 4.6. Inhibition of self-mediated and Cu^2+^ induced Aβ_1-42_ aggregation

Among all the methods to measure the aggregation of Aβ_1-42_, the assay based on Thioflavin T (ThT) is the standard procedure based on the fluorescence of this dye^49^^,^^50^. This test is performed with Aβ_1-42_ peptide previously prepared by dissolving it in 1,1,1,3,3,3–hexafluoro-2-propanol (HFIP), an organic solvent useful to solubilise and monomerize the β–sheet protein aggregates and reserve them in the fridge. Films are re-dissolved in an Eppendorf tube with a fresh solution of a mixture of CH_3_CN/Na_2_CO_3_/NaOH, to have a stable stock solution, and afterwards, added to an optimised fibrillation phosphate buffer^14^. The samples were prepared using MeOH (1 mg/mL) as solvent, followed by incubation in a water bath for 24 h at 37 °C with gentle shaking. After incubation, the samples were added to a 96-well plate (BD Falcon) with 180 µL of 5 µM ThT in 50 mM glycine-NaOH (pH 8.5) buffer. Blank samples were prepared for each concentration in a similar way, devoid of peptide. After 5-min incubation with the dye, the ThT fluorescence was measured at 446 nm (excitation) and 485 nm (emission).

### 4.7. Transmission electron microscopy (TEM)

The Transmission Electron Microscopy (TEM) is a high resolution technique used to study biological or inorganic samples at 2–3 nm level, through black and white high-quality images. The compound **21** was selected among those with better anti-Aβ aggregation assay. Imaging acquisition was performed with samples of the ligand mixed with Aβ, in the absence or presence of copper, prepared by following the single–droplet method in the negative staining protocol. The Aβ stock solutions were prepared by dissolving the lyophilised peptide in a mixture of 48 µL of CH_3_CN, 10 µL of NH_4_OH (2%) and 48 µL of NaCl (300 µM), followed by addition of a buffered solution containing 4–(2-hydroxyethyl)-1-piperazine-ethanesulfonic acid (HEPES, 50 mM, pH = 6.6) to obtain a total concentration of 50 µM. The ligands were treated with amyloid-β peptide and prepared previously to be studied in absence or in presence of copper chloride (25 µM) and everything was followed by incubation for 24 h at 37 °C. Formvar/Carbon 200-mesh Cu grids (Ted Pella) were treated with Aβ peptide aggregated samples (10 µL) for 2 min at rt. Excess sample solution was removed using filter paper followed by washing twice with deionised water. Each grid incubated with uranyl acetate (1%, 10 µL, 1 min) was stained and dried for 15 min at rt. The support device used to hold the sample inside TEM was a copolymer of polyvinyl acetate from polyvnyl alcohol and formaldehyde called *Formvar* stabilised with a thin layer of carbon, using copper 200 – mesh grids as a main support (purchased to Ted Pella).

### 4.8. Cell viability and neuroprotection

SH-SY5Y human neuroblastoma cell line (ATCC-CRL-2266) is grown in Dulbecco’s modified Eagle’s medium (DMEM), and were obtained from Gibco-Invitrogen (Life Technologies Ltd, UK) with 10% heat inactivated foetal calf serum, containing 50 U/mL penicillin and 50 µg/mL streptomycin, under a humidified atmosphere of 95% air- 5% CO_2_ at 37 °C. Cells were plated at 0.12 × 10^6^ cells/mL for cell viability assay. The tested compounds (**18, 19, 20, 21, 23** and **24)** were dissolved in DMSO at a concentration of 50 mM and aliquots were stored at −20 °C. We performed a dose-response screening (from 10 µM to 40 µM) in order to choose the highest non-toxic concentration. As a result we selected: 20 µM final concentrations for compounds **18, 19, 20** and **23**; 20 µM final concentration for compound **19**; 35 µM final concentration for compounds **21** and **24**. The final concentration of DMSO in culture media did not exceed 0.05% (v/v) and no alterations on cells were observed. The compounds were added to the cell media 1 h before the incubation with Aβ_1-42_ or L-ascorbic acid/ferrous sulphate. Aβ_1-42_ or L-ascorbic acid/ferrous sulphate where incubated alone or with the compound for an additional 24 h. Aβ_1-42_ was prepared as 276.9 µM stock in sterile water and added to the medium at 2.5 µM final concentration. Ferrous sulphate was freshly prepared as 0.36 M stock in water and added to the medium at 500 µM final concentration. *L*-Ascorbic Acid was freshly prepared as 80 mM stock in water and added to the medium at 5 mM final concentration. Aβ_1-42_ was purchased from Bachem (Torrance, CA, USA) and ferrous sulphate and L-ascorbic acid from Sigma Chemical Co (St. Louis, MO, USA).

Cell viability was determined by the MTT (3–(4,5-dimethylthiazol-2-yl)-2,5-diphenyltetrazolium bromide) reduction test. In viable cells, the enzyme succinate dehydrogenase metabolises MTT into a formazan that absorbs light at 570 nm. Following the cell treatment protocol, the medium was aspirated and 0.5 mL MTT (0.5 mg/mL) was added to each well. The plate was then incubated at 37 °C for 1 h 30 min protected from light. At the end of the incubation period, the formazan precipitates were solubilised with 0.5 mL of acidic isopropanol (0.04 M HCl/isopropanol). The absorbance was measured at 570 nm^51^. Cell reduction ability was expressed as a percentage of untreated control cells.

All data were expressed as mean ± SEM of at least three independent experiments performed in duplicates. Statistical analyses were performed using GraphPad Prism 5 (GraphPad Software, San Diego, CA, USA). Differences between two datasets were evaluated by two-tailed unpaired Student’s t test A *p*-value <0.05 was considered statistically significant.
